# Extensive Multicystic Ameloblastoma of the Mandible Managed by Hemimandibulectomy and Immediate Reconstruction: A Case Report

**DOI:** 10.7759/cureus.92468

**Published:** 2025-09-16

**Authors:** Andrianos Tzortzis, Ourania Tzortzi, Konstantinos Stivaktakis, George Tzortzis

**Affiliations:** 1 General Surgery, Antrim Area Hospital, Northern Health and Social Care Trust, Antrim, GBR; 2 School of Dentistry, National and Kapodistrian University of Athens, Athens, GRC; 3 ENT, Panarcadian General Hospital of Tripolis, Tripolis, GRC; 4 Oral and Maxillofacial Surgery, Panarcadian General Hospital of Tripolis, Tripolis, GRC

**Keywords:** ameloblastoma, facial reconstruction, hemimandibulectomy, odontogenic tumours, treatment

## Abstract

Ameloblastoma is one of the most common odontogenic tumors. It usually has slow growth and presents as an asymptomatic swelling. The diagnosis depends on imaging and histological examination, and the current management consists of surgical excision with restoration of the functionality and the aesthetics of the area. In this paper, we present the management of a 53-year-old man with an extensive ameloblastoma of the mandible, which measured 15.5x8.2x9.6 cm. The patient underwent hemi-mandibulectomy, followed by placement of a reconstruction plate. This one-stage treatment established early functioning and aesthetic recovery for the patient. The patient was followed up and has remained asymptomatic for 24 months after the operation.

## Introduction

Ameloblastoma is one of the most common odontogenic tumors. It is a benign but locally invasive tumor that originates from the odontogenic epithelium. However, the odontogenic ectomesenchyme is not involved. It comprises approximately 1-3% of the tumors of the jaws and is mostly found in the mandible (80%), compared to the maxilla (20%) [1]. However, malignant ameloblastoma, including metastasizing ameloblastoma and ameloblastic carcinoma, is extremely rare, and it comprises approximately 1% of ameloblastoma cases [2,3].

Ameloblastoma is characterized by slow growth and presents as an asymptomatic swelling, resulting in a delay in its diagnosis, and if neglected, it causes severe deformity of the face [4,5]. The diagnosis relies on imaging and histological examination. The treatment consists of surgical excision with restoration of the functionality and the aesthetics of the area. This can be achieved with more conservative methods, such as curettage or enucleation, or with more aggressive techniques, like marginal or segmental resection [4,5].

In this paper, we present the management of a 53-year-old male patient with an extensive ameloblastoma of the mandible, who underwent hemi-mandibulectomy.

This work was previously presented at the HAOMS 2025 meeting on January 26, 2025.

## Case presentation

A 53-year-old man presented to the Oral and Maxillofacial Department with a swelling on the left side of his mandible, extending to the ipsilateral side of his neck. He was diagnosed with mandibular ameloblastoma eight years ago, but he did not pursue treatment at that time. His past medical history was unremarkable.

**Figure 1:**
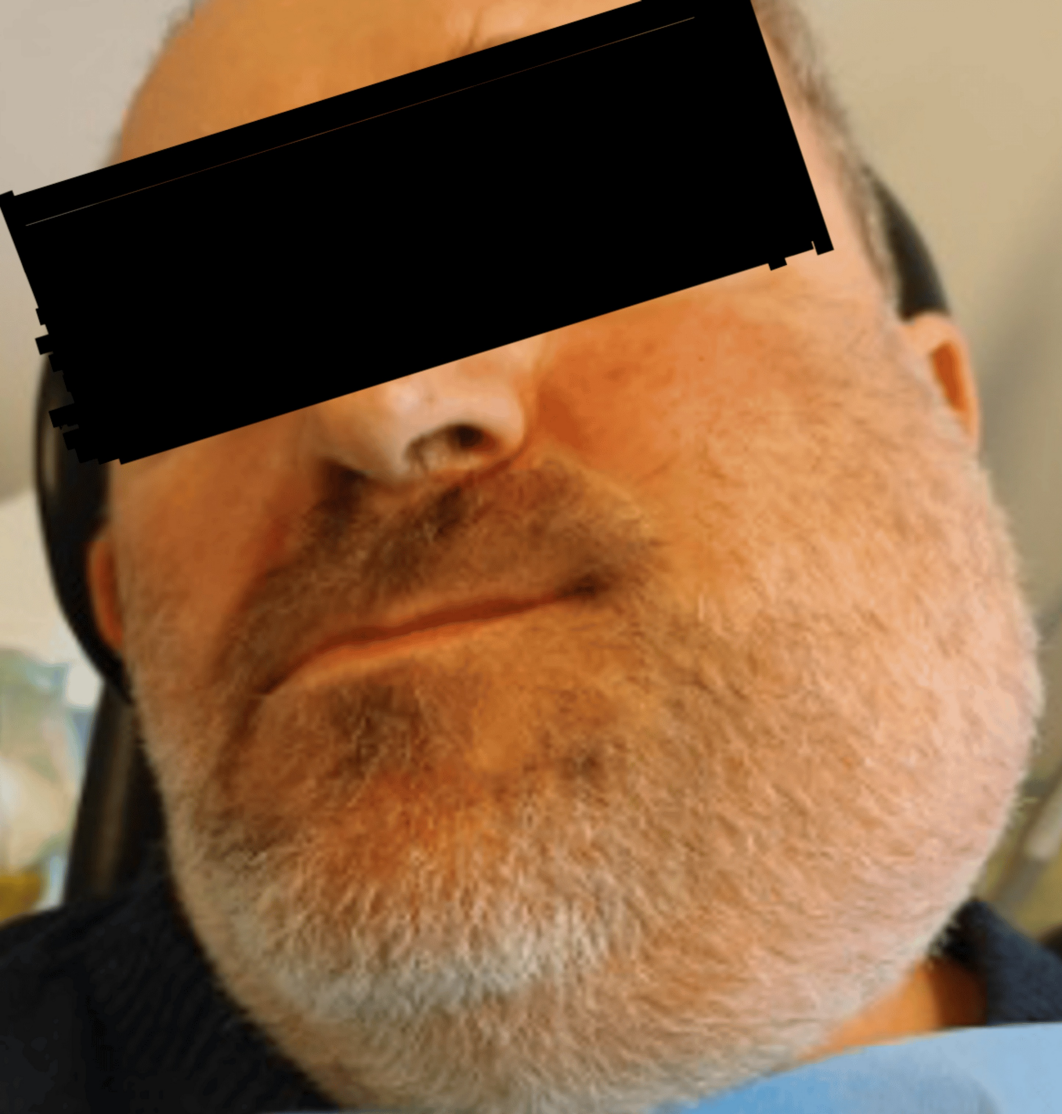
Preoperative facial appearance

**Figure 2:**
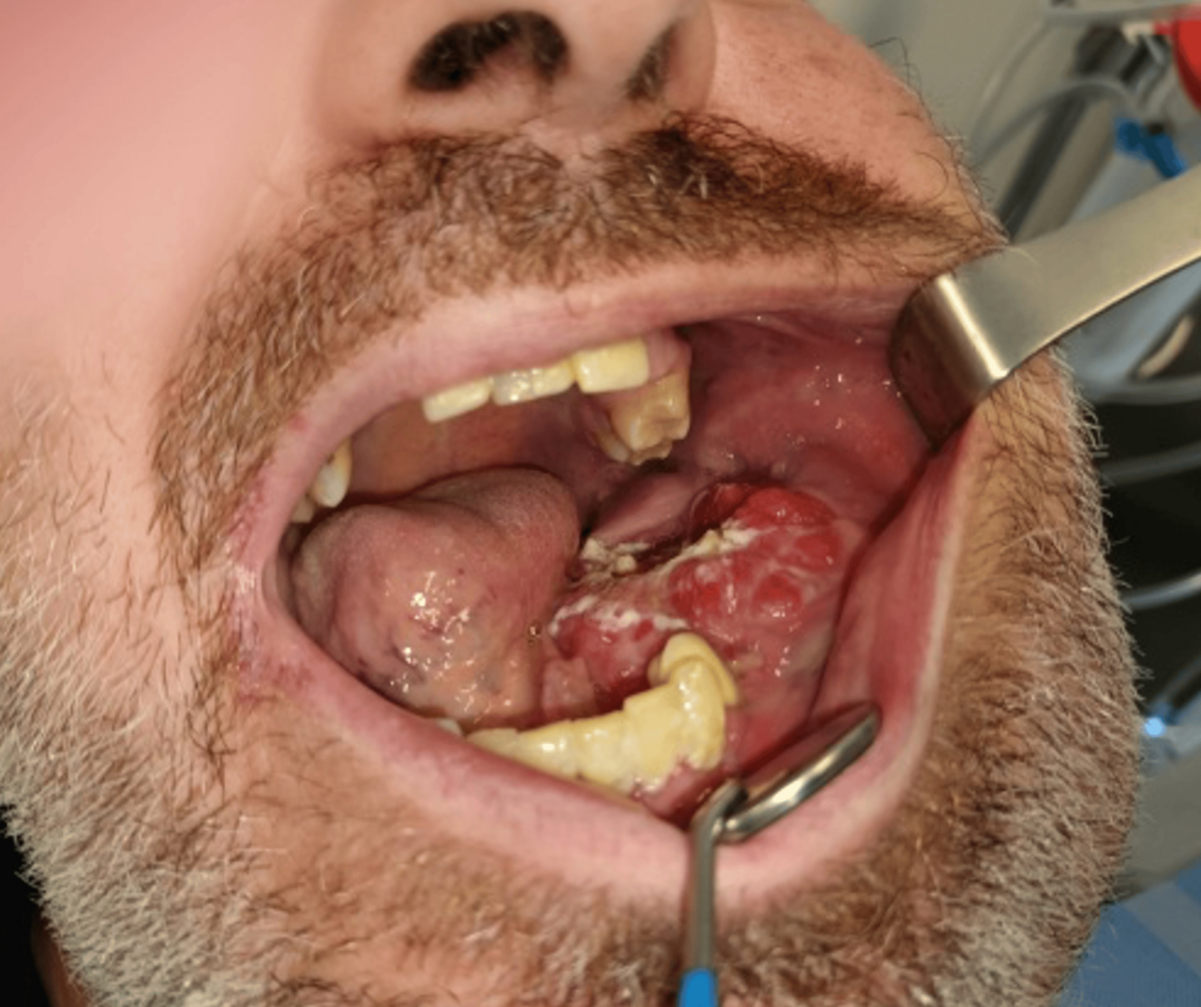
Preoperative intra-oral view showing erosion of the oral mucosa

On the clinical examination, there was a firm mass extending from the midline to the condyle of the mandible, associated with erosion of the oral mucosa and no palpable lymph nodes (Figures 1, 2).

There were poor oral hygiene and paresthesia in the area of distribution of the inferior alveolar nerve.

**Figure 3:**
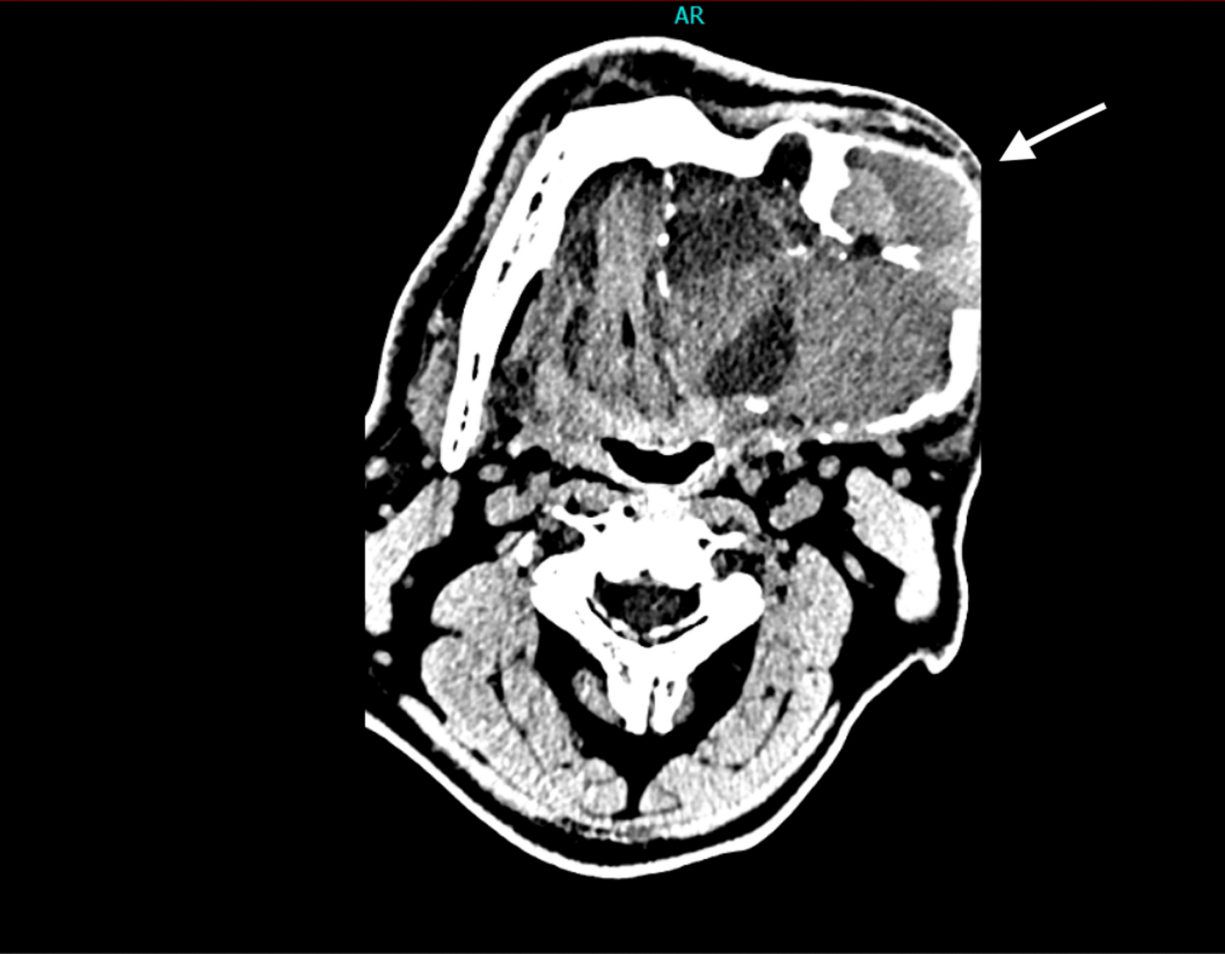
CT axial section showing extension of the lesion and both buccal and lingual cortical plate expansion and thinning (arrow)

**Figure 4:**
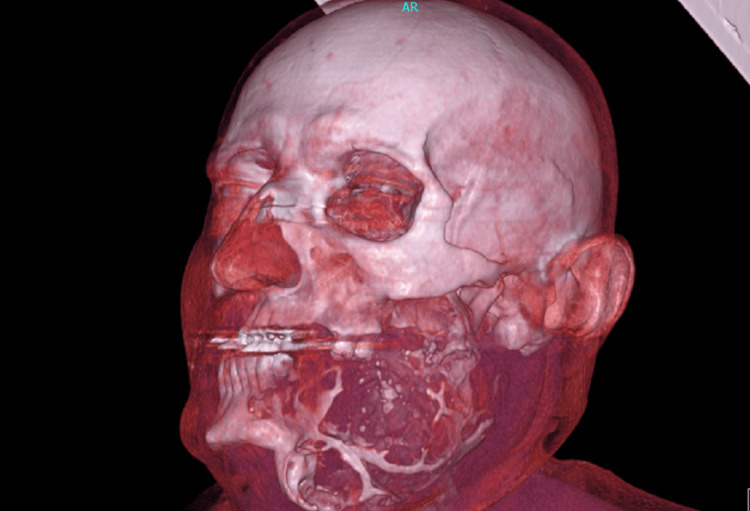
3D reconstruction showing destruction and erosion of the left mandible

**Figure 5:**
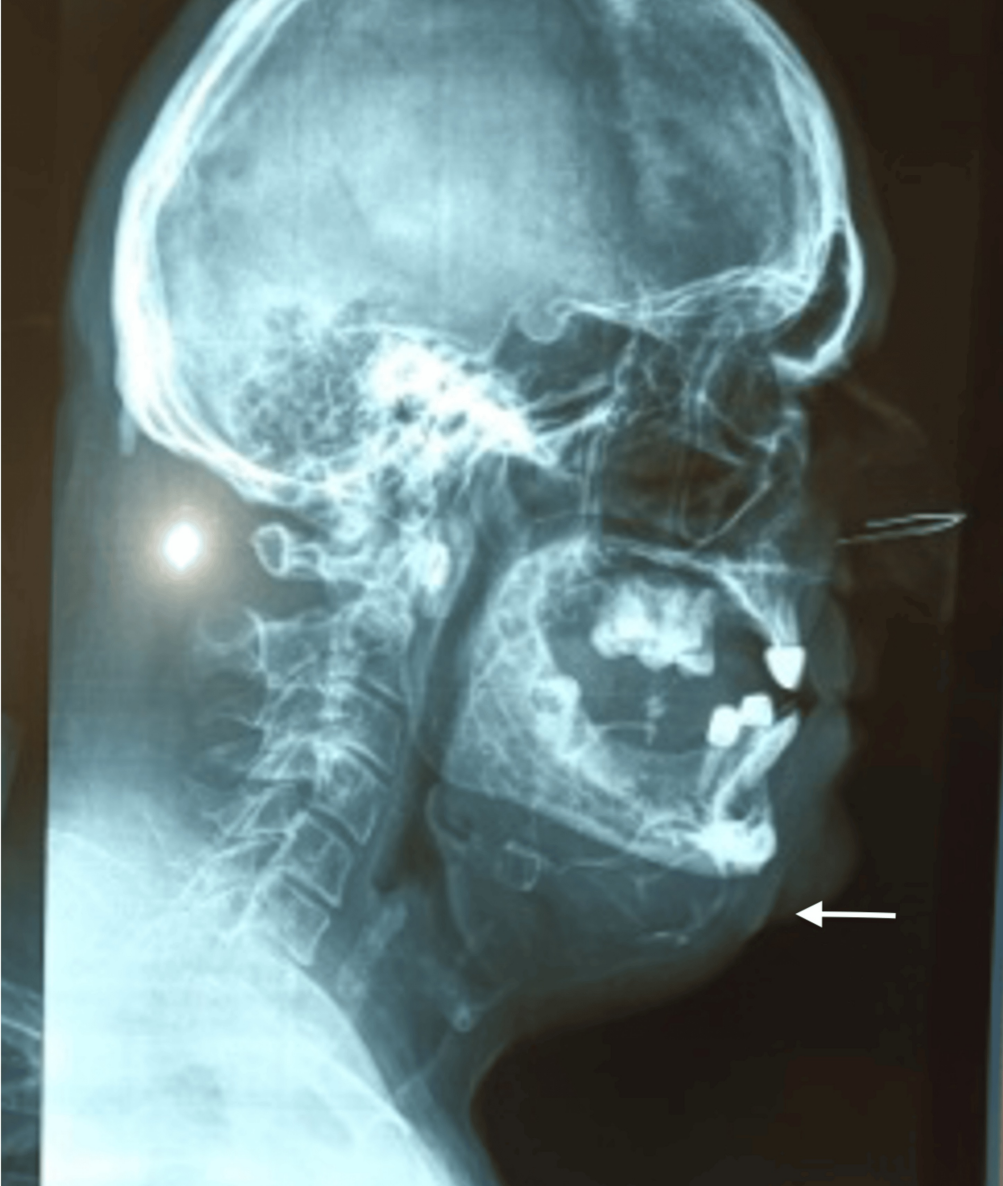
Lateral view: plain X-ray film showing a soap bubble-type pattern

The radiological exams included computed tomography (CT), cone beam computed tomography (CBCT), and plain X-ray films. The radiographic examination showed a multilocular lesion with expansion and thinning of the bony plates, and with the characteristic "soap bubble" appearance on plain radiographs. The size of the tumor was 15.5x8.2x9.6 cm. Based on the clinical and radiographic features of the lesion, the suspicion was that of a solid or a multi-cystic ameloblastoma (Figures 3-5).

**Figure 6:**
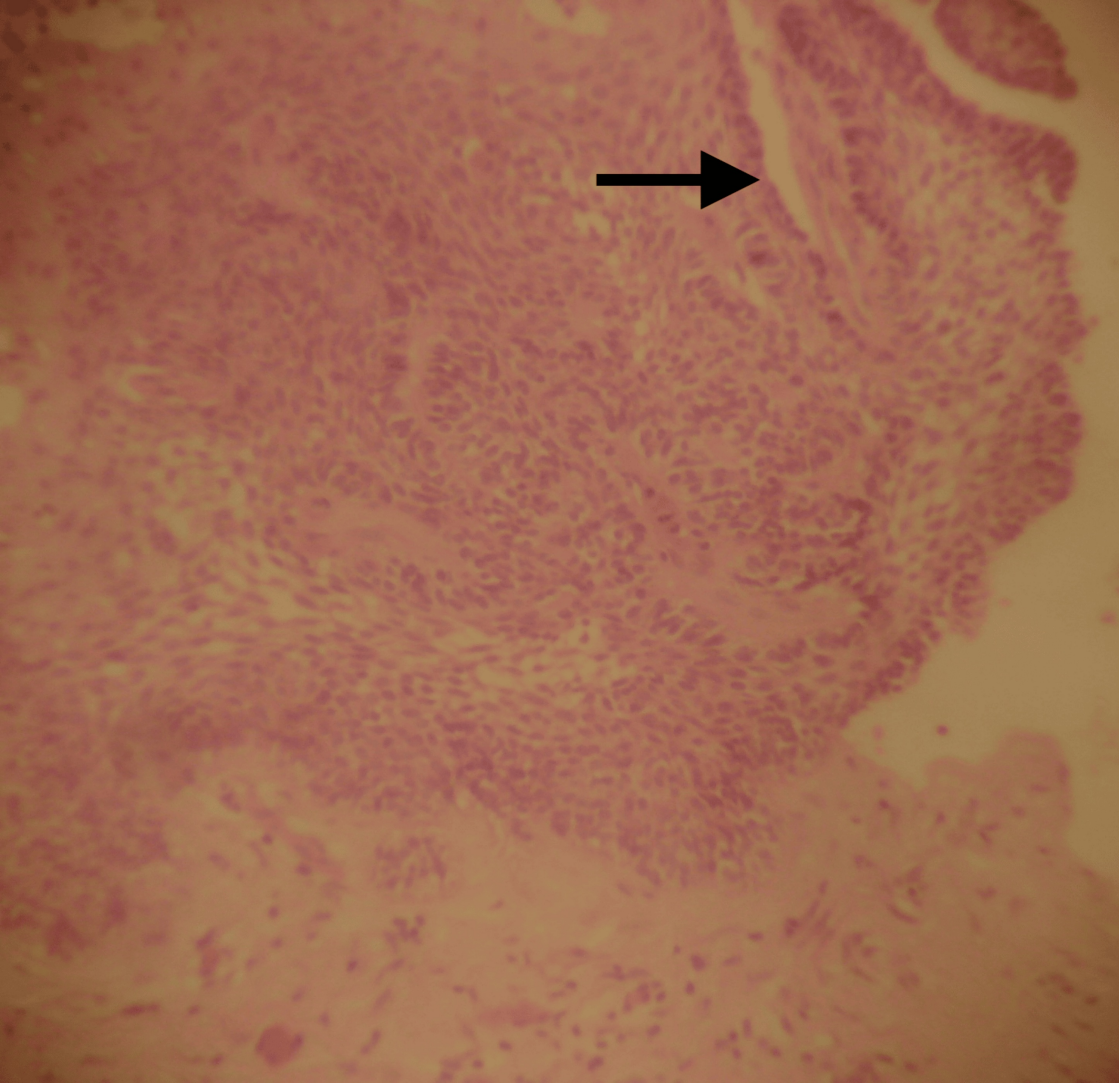
Histological picture showing mixed follicular-acanthomatous-type ameloblastoma with extended squamous metaplasia and focal keratin formation (arrow) within central portions of the epithelial follicular formations (H&E X20)

The patient underwent further biopsy under local anesthesia, and tissue was collected both intra- and extraorally. Histological examination revealed a mixed follicular-acanthomatous type, showing epithelial islands resembling the enamel organ, surrounded by a peripheral layer of typical ameloblasts, within a background of dense fibrous connective tissue. In addition, there was extensive squamous metaplasia, along with keratin formation in the central areas of the epithelial follicular structures (Figure 6).

The patient was taken to the operating theatre after appropriate clinical and laboratory evaluation. Initially, nasotracheal intubation was performed, a nasogastric tube was placed, and at the end of the procedure, tracheostomy was performed.

**Figure 7:**
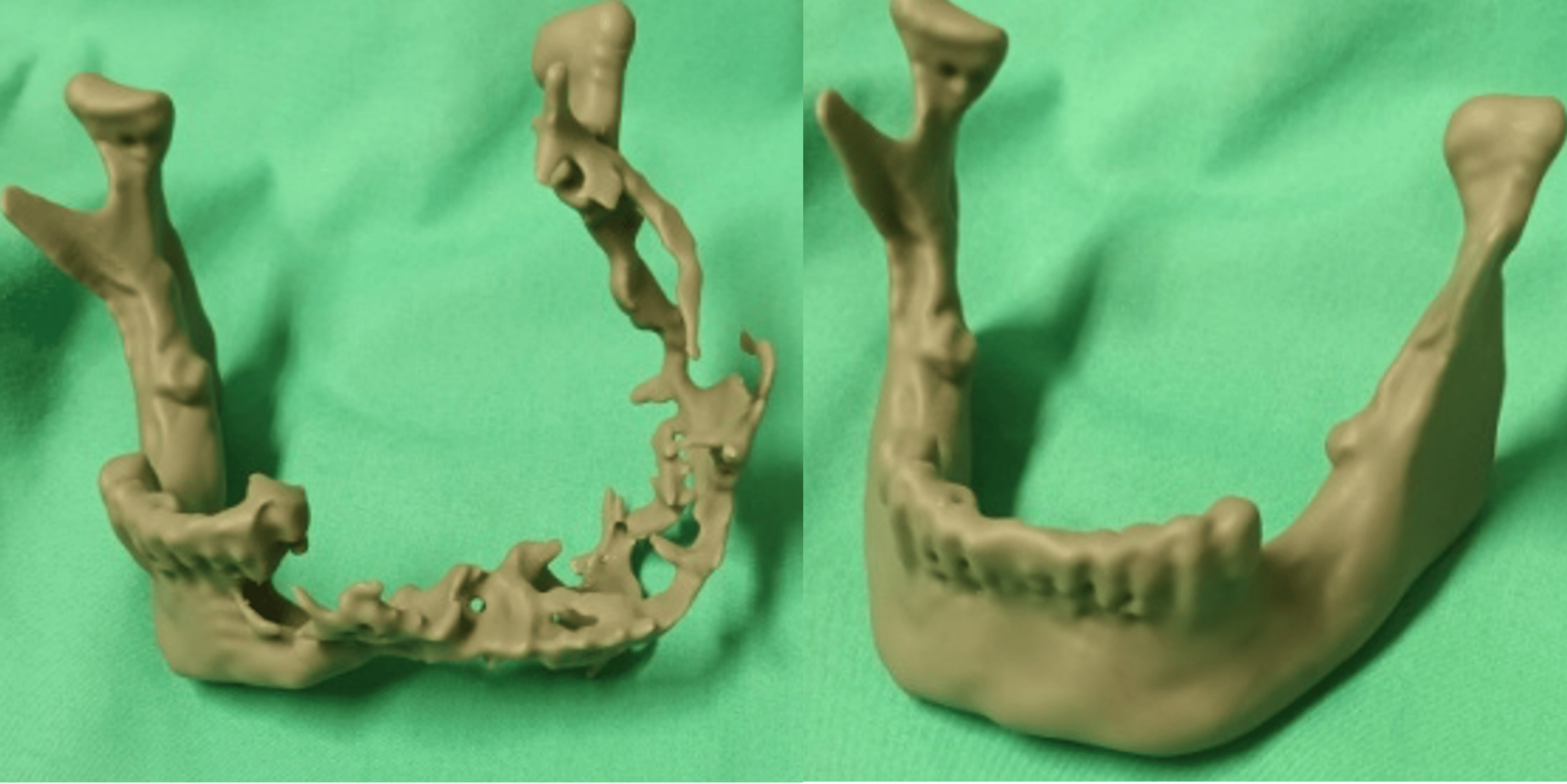
Stereolithographic model of the mandible used for modeling of a reconstructive plate with condyle preoperatively

A stereolithographic 3D model designed preoperatively played an important role in planning both the resection and the shaping of the reconstruction plate, which was placed intraoperatively (Figure 7).

**Figure 8:**
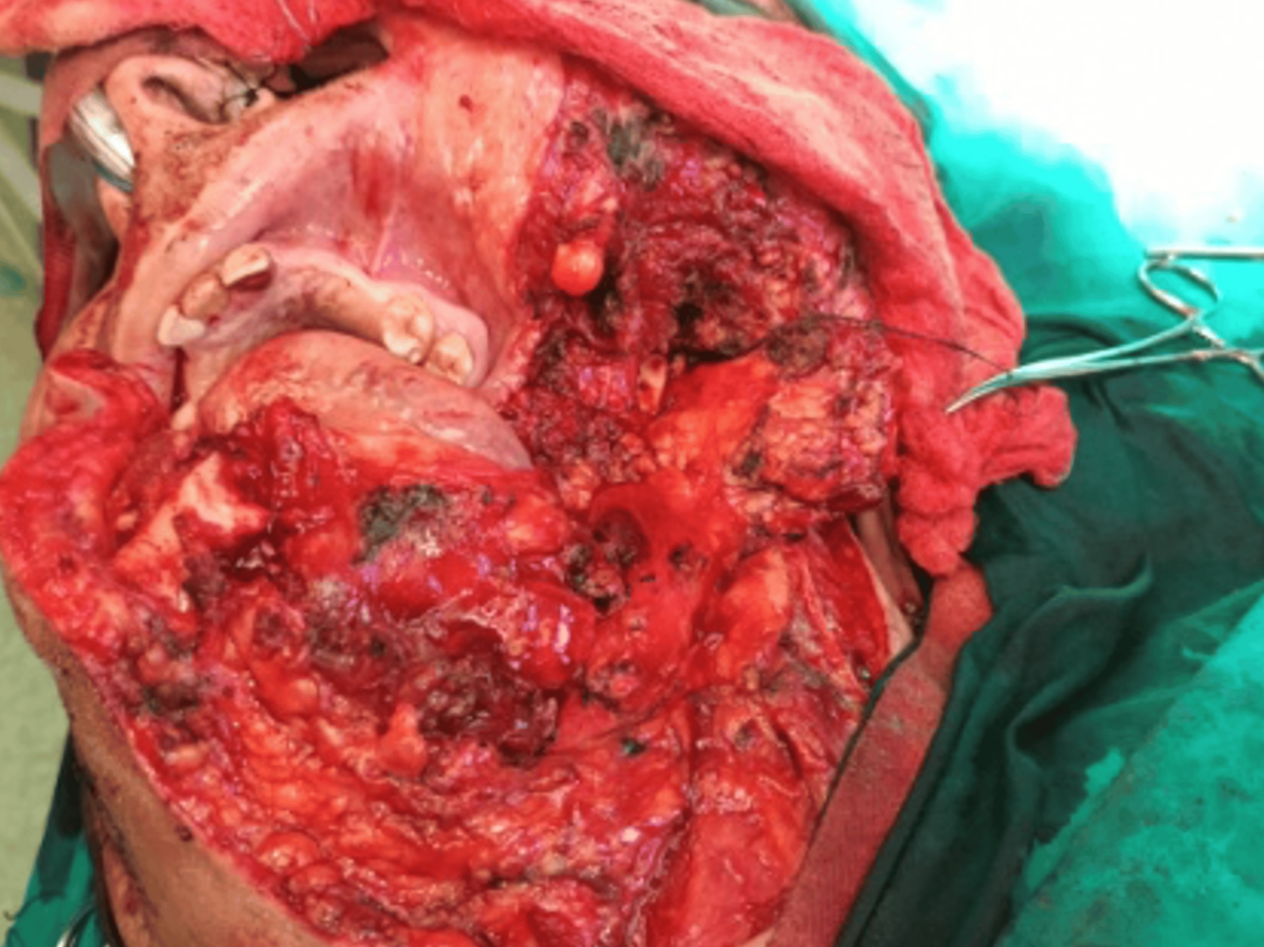
Intraoperative picture showing the surgical defect through the extra-oral approach and hemimandibulectomy

**Figure 9:**
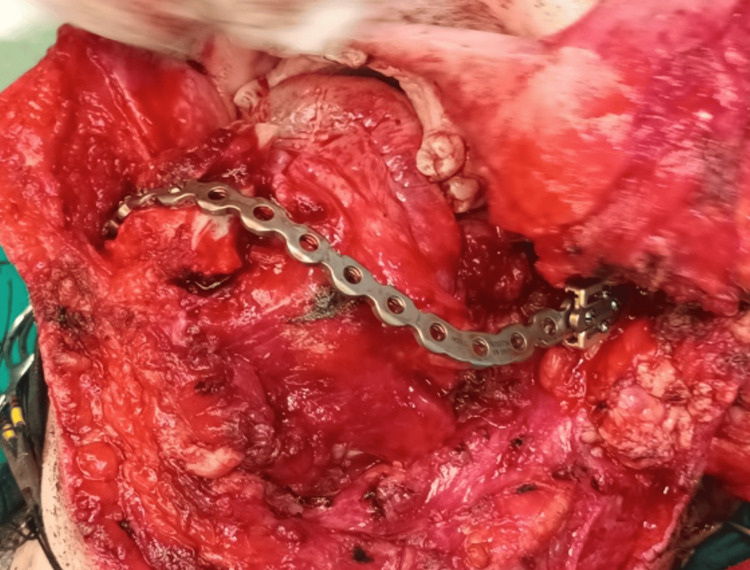
Intraoperative image showing the immediate reconstruction with a titanium plate and condyle

Due to the extensive infiltration of the lesion into the oral mucosa, the surgical plan included harvesting a pectoralis major flap in case of a large tissue defect. A submandibular approach was performed via a low incision in the neck, extending from the midline of the lower lip to the mastoid process, with splitting of the lip allowing for a wide surgical field. Resection was carried out from the midline of the mandible, including removal of the ipsilateral condyle with a safety margin of approximately 1.5 cm, along with the surrounding affected soft tissues. Following the resection, a titanium reconstruction plate with condylar components was placed. Intraoral suturing was performed very carefully and without tension, and there was no need for flap harvesting. The skin flap was sutured in layers (Figures 8, 9).

**Figure 10:**
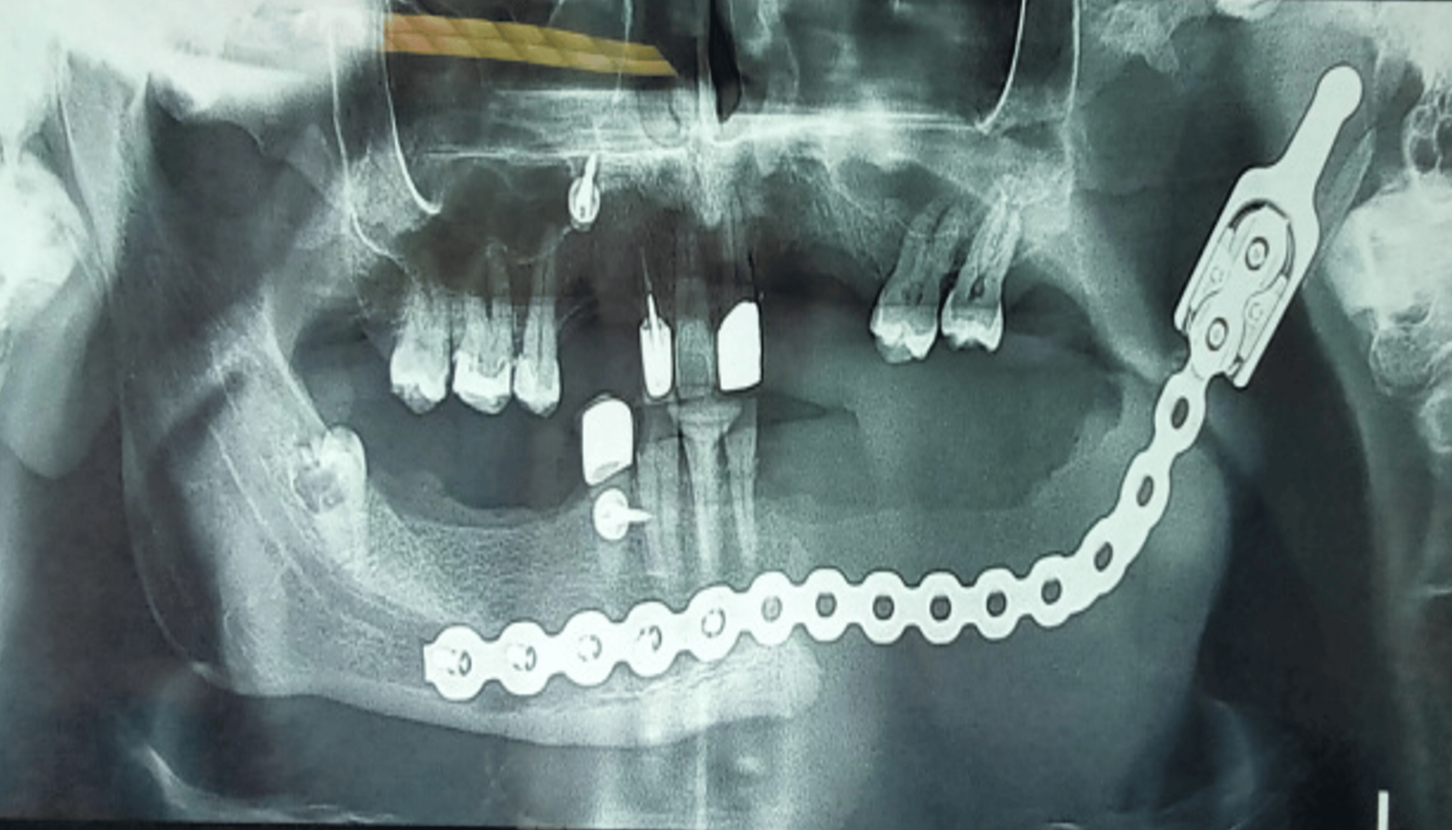
Postoperative orthopantogram (OPT) showing mandibular reconstruction with a titanium plate and a condyle component

**Figure 11:**
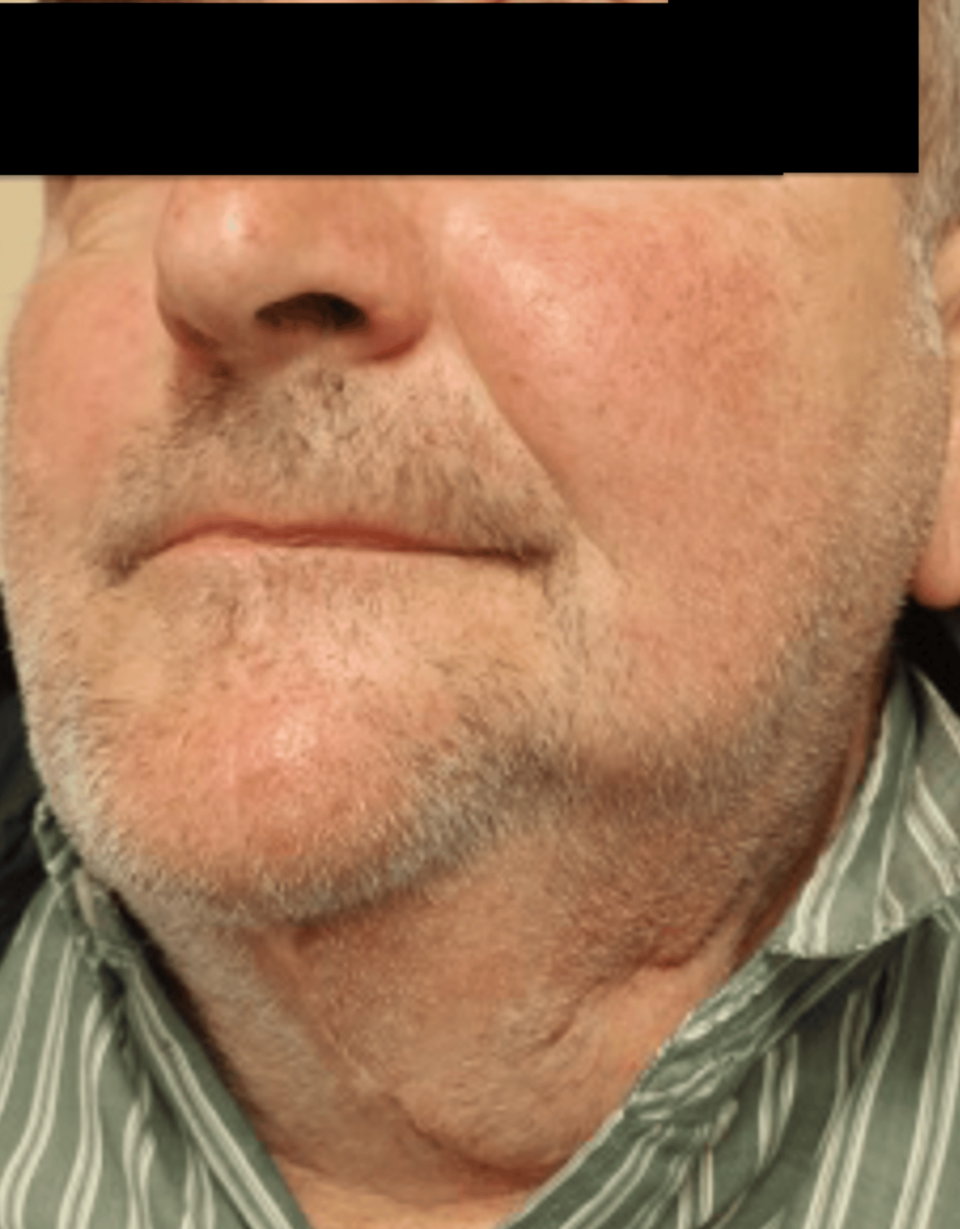
Facial appearance six months postoperatively, showing satisfactory cosmetic results and healing

The patient remained in the hospital for a total of 14 days, initially for two the ICU and then in a regular ward. His postoperative course was satisfactory, and he underwent a postoperative orthopantogram (Figure 10). He is now being followed up in the clinic at regular intervals (Figure 11). His mastication, speech and quality of life have returned to his previous baseline, with no recurrence of the disease.

## Discussion

Ameloblastoma is considered a locally aggressive tumor, with its therapeutic approach being individualized and determined by a combination of factors, such as its anatomical location, clinical and radiographic type, histological subtype, radiographic appearance, and the size of the lesion. There are three different clinicopathological types of ameloblastoma: conventional (solid or multicystic), unicystic, and peripheral (extraosseous) ameloblastoma [6,7].

Preoperative CT scanning provides information through both bone and soft tissue windows, playing a crucial role in surgical planning. Ameloblastomas that appear as multilocular radiolucencies with thinning and erosion of the bone plates are considered more aggressive than those presenting as unilocular radiolucencies [8].

The solid or multicystic ameloblastoma, such as the case we present here, typically appears in individuals from the third to the sixth decade of life. It usually presents as a painless swelling of the jaw, which, if left untreated, continues to grow slowly and can reach considerable dimensions, potentially causing facial deformity. In our case, the patient had been diagnosed with ameloblastoma eight years earlier, without having received treatment [9,10].

To date, many histological types of solid or multicystic ameloblastoma have been identified. Combinations of histological types are often observed. In our case, the histological examination showed a mixed follicular-acanthomatous type. The follicular type is the most common histological form, whereas the acanthomatous type is characterized by extensive squamous metaplasia. The combination of the two does not indicate more aggressive behavior. However, histologically, there can be diagnostic confusion with squamous cell carcinoma or squamous odontogenic tumor [11].

The treatment of choice is surgical resection. The choice of the appropriate surgical strategy is key to successful management. Conservative approaches include enucleation or curettage. These are recommended for unilocular, peripheral, and generally smaller lesions. However, because small islands of the tumor often remain in the surgical field in cases of conservative treatment, the recurrence rate can reach 55-90 %. Radical treatment usually involves segmental resection of the affected bone and any involved soft tissues, with a margin of 1-2 cm beyond the lesion. The recurrence rate in cases treated with radical resection is 15-25%. In our case, radical resection was selected. A hemimandibulectomy was performed, along with removal of the affected soft tissues with clear margins of approximately 1.5 cm [4,5].

The surgical defect after the resection can be managed either during the same operation or later, using autologous or heterologous grafts, with the use of free flaps, as well as a variety of alloplastic materials and allografts. In our case, the patient was provided with both options, but he was not keen to have reconstruction with the use of a flap. Hence, a pre-designed titanium reconstruction plate with a condyle component was used to reconstruct the bone defect. A flap was not used for the soft tissue defect, as layered, airtight suturing was successfully achieved [5,12].

Nowadays, the view is gaining ground that the development and progression of odontogenic tumors is due to genetic and molecular alterations. Therefore, further molecular studies are needed to clarify the etiopathogenesis of odontogenic tumors and to develop new therapeutic options, such as targeted molecular therapy, as alternatives or adjuncts to existing surgical methods [1,13].

## Conclusions

Ameloblastoma is one of the most common odontogenic tumors. In this paper, we presented a case of an extended ameloblastoma that was diagnosed eight years ago and was not treated at the time of diagnosis. The treatment should be tailored to each case, taking into consideration the clinical, radiological, and histological characteristics of the tumor. The balance between appropriate surgical technique and a low recurrence rate has been challenging to achieve. Radical removal of the tumor is associated with lower recurrence rates compared to more conservative management. In our case, we performed a hemimandibulectomy and immediate reconstruction to achieve a better aesthetic and functioning outcome.

The patient should be followed up for many years after the operation, for continuous monitoring of recurrence. Molecular characteristics of these tumors could play an important role in the personalized management of each case. More studies that assess the molecular profile of ameloblastomas are needed.
